# Probing photoprotection properties of lipophilic chain conjugated thiourea-aryl group molecules to attenuate ultraviolet-A induced cellular and DNA damages

**DOI:** 10.1038/s41598-022-25515-5

**Published:** 2022-12-03

**Authors:** Sobia Rana, Noor Fatima, Sana Yaqoob, Abdul Hameed, Munazza Raza Mirza, Almas Jabeen, Jamshed Iqbal

**Affiliations:** 1 Dr. Panjwani Center for Molecular Medicine and Drug Research (PCMD), International Center for Chemical and Biological Sciences (ICCBS), University of Karachi, Karachi, 75270 Pakistan; 2 H. E. J. Research Institute of Chemistry, International Center for Chemical and Biological Sciences, University of Karachi, Karachi, 75270 Pakistan; 3 Department of Chemistry, University of Sahiwal, Sahiwal, Pakistan; 4 Center for Advanced Drug Research, COMSATS University Islamabad, Abbottabad Campus, Abbottabad, 22060 Pakistan

**Keywords:** Cell biology, Molecular biology

## Abstract

Ultraviolet-A (UVA) radiation is a major contributor to reactive oxygen species (ROS), reactive nitrite species (RNS), inflammation, and DNA damage, which causes photoaging and photocarcinogenesis. This study aimed to evaluate the UVA protective potential of lipophilic chain conjugated thiourea-substituted aryl group molecules against UVA-induced cellular damages in human dermal fibroblasts (BJ cell line). We tested a series of nineteen (19) molecules for UVA photoprotection, from which 2′,5′-dichlorophenyl-substituted molecule **DD-04** showed remarkable UVA protection properties compared to the reference (benzophenone). The results indicate that **DD-04** significantly reduced intracellular ROS and nitric oxide (NO) as compared to the UVA-irradiated control (*p* < 0.001). Moreover, the compound **DD-04** showed anti-inflammatory activity as it significantly reduced the levels of tumor necrosis factor-α (TNF-α) and interleukin-1β (IL-1β) pro-inflammatory cytokines produced by THP-1 (human monocytic) cells (*p* < 0.05). DNA damage was also prevented by **DD-04** treatment in the presence of UVA. It was observed that **DD-04** significantly reduced the number of cyclobutane pyrimidine dimers (CPDs) when compared to the UVA-irradiated control (*p* < 0.001). Finally, the DNA strand breaks were checked and a single intact DNA band was seen upon treatment with **DD-04** in the presence of UVA. In conclusion, **DD-04** can be considered a potential candidate UVA filter due to its photoprotective potential.

## Introduction

The solar ultraviolet (UV) radiation-induced damages are the major environmental risk factors leading to various skin-related diseases including pre-mature photoaging and photocarcinogenesis^1^. UVA is the most abundant solar UV radiation and comprises greater than 90% of the total terrestrial solar UV radiation and is 1.6 times more penetrable than UVB^2,3^. Cloud cover decreases the intensity of UVB radiation to some extent but UVA radiation easily passes through the clouds and even glass^4^. Therefore, the lifetime exposure of an individual to UVA rays is much higher than that of UVB rays^5^. UVA was once thought to be innocuous but both natural and artificial source of UVA elevates the level of reactive oxygen species (ROS), reactive nitrite species (RNS), inflammation, and DNA damage^6^. UVA easily reaches the deeper layer of skin, the dermis, which is rich in extracellular matrix (ECM) producing fibroblasts and other types of cells including macrophages, adipocytes, mast cells, and stem cells^7,8^. The ECM is vital for skin architecture, physiology, and wound healing^9^. UVA is responsible for the upregulation of the matrix metalloproteinase (MMP) enzymes which degrades the ECM in the dermis^10,11^. These damages to the ECM result in early wrinkling of the skin and premature skin photoaging^9^.

UV radiation primarily damages the molecular structures within the cell by two mechanisms. The direct mechanism involves the absorption of UV radiation by endogenous chromophores like DNA, urocanic acid, amino acids, melanin, and other metabolites while the indirect mechanism of UV damage requires the interaction of the cellular molecules with ROS and RNS^1,12^. ROS generation is the hallmark of UVA exposure and leads to the oxidation of lipids, proteins, and DNA bases. These damages deteriorate the genomic and cellular integrity, which results in either carcinogenesis or apoptosis^13^. The UVA-induced ROS is also linked to the activation of various transcription factors including the nuclear factor (NF)-κB, which induces the production of tumor necrosis factor-α (TNF-α) and interleukin-1β (IL-1β)^14,15^. NF-κB also upregulates the production of nitric oxide (NO) via the expression of the inducible nitric oxide synthase (iNOS) enzyme which is a potent pro-inflammatory mediator^16,17^.

According to the American Cancer Society, UVA is considered a human carcinogen^18^. The *p53* tumor suppressor gene product regulates various cellular processes including cell cycle arrest, apoptosis, and DNA repair^19^. UVA has been shown to increase the expression of the *p53* gene, which is directly involved in the nucleotide excision repair (NER) of UV-induced cyclobutane pyrimidine dimers (CPDs)^20^. The oncogenic transformation of the *p53* gene is linked to various types of cancer due to the loss of an efficient DNA repair system. A genome mapping study revealed that UVA elicited mutational changes within the region of the *p53* gene by the accumulation of CPDs and oxidative DNA damage^21^. Various in vivo studies suggest that UVA can promote melanomagenesis. In a study, the effect of UVA-induced melanoma formation was compared between black (melanin-containing) and albino (without melanin) transgenic mice. The results of the study revealed that melanoma formation depends upon the presence of melanin pigment as melanoma was formed only in black mice^22^. These findings are quite intriguing as melanin pigment has always been thought to protect from skin carcinogenesis especially in people of color^23^.

CPD or simply the thymine dimers are the most abundant DNA lesion formed after UVA exposure^24^. A study showed that UVA causes damage to DNA in a ratio of 1:1:3:10 for oxidized pyrimidines, single-strand breaks, oxidized purines, and CPD, respectively. Although UVA immensely generates ROS/RNS, the studies indicate that UVA predominantly generates CPD rather than oxidized bases like 8-oxoG^25,26^. These CPDs are responsible for the inhibition of DNA replication as it is formed by covalently bonded adjacent thymine on the same strand of DNA. CPD removal in mammals is solely dependent on the NER system and if not removed it may mutate genes associated with the regulation of cell proliferation (*p53* gene)^27^. Moreover, UVA-induced strand breaks in the DNA are also highly mutagenic^28^. Single-strand breaks (SSBs) when left unrepaired form double-strand breaks (DSBs) via polymerase enzyme. These DSBs are deleterious to the cells and cause chromosomal rearrangements and initiation of skin malignancies especially when the stem cell compartment of the dermis is damaged^24^. Like CPDs, DNA strand breaks are also repaired via activation of the *p53* gene^29^.

**Figure 1:**
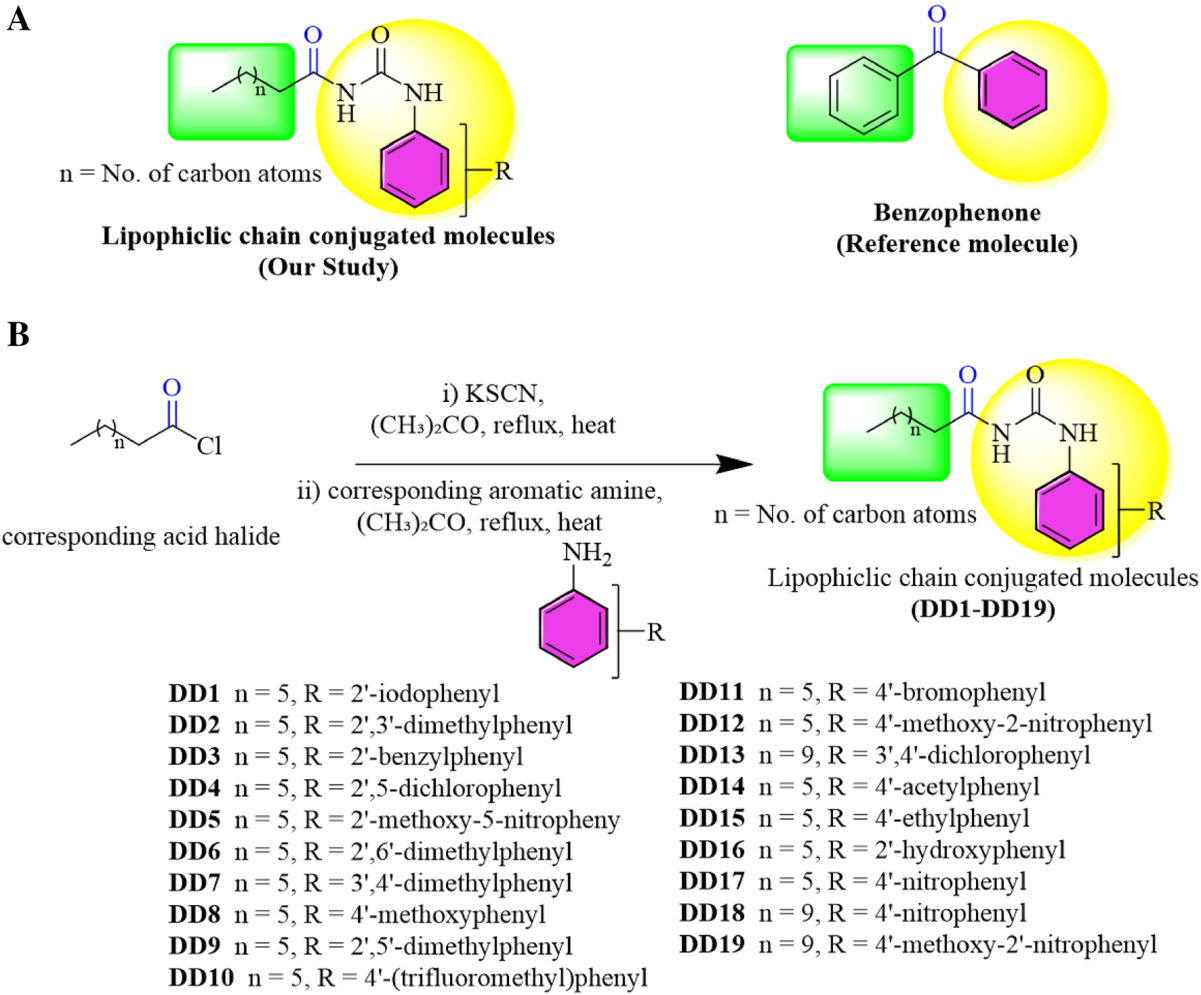
Structures of lipophilic chain conjugated molecules and reference “benzophenone” (**A**). Synthesis of lipophilic chain linked via thiourea moiety with a substituted aromatic ring containing conjugates (**B**)^34^.

Currently, all of the marketed UVA filters that are being used in sunscreens have many controversies regarding their toxicity and adverse health effects^30^. Therefore, there is a need to develop new photoprotective agents with less toxicity and greater protective potential against UV-induced inflammation, cutaneous malignancies, and photoaging. Thiourea is an organosulfur compound having a diverse range of pharmacological applications in various diseases linked to bacterial infections, oxidative stress, and inflammation^31^. Moreover, it has also been shown that thiourea derivatives pose anti-melanoma activity and it also inhibits the production of MMPs by suppressing TNF-α production^31–33^. Thus, the current study was proposed to elucidate the in vitro cytotoxicity and photoprotection ability of the lipophilic chain-linked thiourea derivatives. The lipophilic derivatives have a close resemblance with the reference molecule due to the presence of an aromatic ring on one side and an alkyl chain on the other linked via a carbonyl moiety (Fig. 1A). To our knowledge, thiourea derivatives have not yet been studied for their UVA protective potential.

## Methods

### Chemistry

The lipophilic chain conjugates with substituted aromatic rings via thiourea moiety have been synthesized from corresponding acid halide (Fig. 1B)^34^.

### Bioactivity

#### Cell lines and cell culture maintenance

The Biobank facility of ICCBS, University of Karachi, provided the normal human dermal fibroblasts cell line, BJ (CRL-2522) obtained from the ATCC (American Type Culture Collection, USA), and the human leukemia monocytic cell line (THP-1) which was purchased from the ECACC (European Collection of Cell Cultures, UK). The BJ cells were cultured in Dulbecco's Modified Eagle Medium/Nutrient Mixture F-12 media (DMEM/F-12; Thermo Fisher Scientific, USA) supplemented with 10% heat-inactivated fetal bovine serum (FBS; Thermo Fisher Scientific, USA), 1% antibiotics (100 unit/mL of penicillin and 100 µg/mL of streptomycin; Thermo Fisher Scientific, USA), and 1 mM sodium pyruvate (Thermo Fisher Scientific, USA). The THP-1 cell line was maintained in RPMI 1640 medium (Thermo Fisher Scientific, USA) with 10% FBS and antibiotics as mentioned earlier. Both the cell lines were maintained in a humidified incubator at 37 °C with 5% CO_2_.

#### Measurement of cytotoxicity (IC_50_)

Cells were seeded in a 96-well plate at a density of 1 × 10^4^ cells per well for the measurement of cytotoxicity of the compounds (without UVA irradiation). Following 24 h of incubation, the cells were treated with various concentrations (10 µM, 25 µM, 50 µM, and 100 µM) of both the reference compound benzophenone (BP; Fluka, Switzerland) and the test compounds (DD-01 to DD-19). Cells after treatment were incubated for 48 h. The cell viability was determined by colorimetric 3-(4,5-dimethyl thiazolyl-2)-2,5-diphenyltetrazolium bromide (MTT; Thermo Fisher Scientific, USA) assay. Briefly, 0.5 mg/mL MTT solution was dispensed in each well of the 96-well plate and incubated for 4 h. Afterward, the MTT solution was removed and 200 µL of 100% dimethyl sulfoxide (DMSO; Thermo Fisher Scientific, USA) was added to each well and the plate was placed on a microplate shaker for 5 min to dissolve the formazan granules. The absorbance was measured at 550 nm using a spectrophotometer (Multiskan GO Spectrophotometer, Thermo Fisher Scientific, MA, USA).

#### Optimization of UVA irradiation dose

The UVA irradiation was carried out using a UV lamp (Uvitec, Cambridge, UK) equipped with a fluorescent bulb emitting a peak 365 nm wavelength. The BJ cells were seeded in a 96-well plate and kept in an incubator for 24 h. The growth medium was replaced with PBS prior to UVA irradiation. Cells were exposed to different doses of UVA (5 kJ/cm^2^, 10 kJ/cm^2^, 15 kJ/cm^2^, 20 kJ/cm^2^ and 30 kJ/cm^2^). Afterward, PBS was replaced with a fresh incomplete medium (media without serum) and cells were incubated further for 24 h. The control cells were not exposed to UVA but were kept under the same experimental conditions as the UVA irradiated cells. Cell viability was determined by MTT assay as mentioned previously.

#### Determination of treatment dose (EC_50_)

The EC_50_ treatment dose was determined by performing a two-fold serial dilution of the IC_50_ concentration (µM) of the selected compounds and the reference compound in sterile PBS. Then, the cells were irradiated with UVA at the selected dose of 30 kJ/cm^2^ in presence of the compounds. After exposure, the incomplete growth medium (DMEM/F-12 without FBS) was added to UVA-irradiated cells with the serially diluted concentrations of each compound in their respective wells as mentioned above. After 24 h MTT assay was performed.

#### Determination of nitric oxide (NO) production

The nitrite (NO^2−^) accumulation in supernatant corresponds to the nitric oxide (NO) production as it is one of the two stable breakdown products of NO. Briefly, the cells were treated with compounds at EC_50_ concentration and exposed to UVA as previously mentioned. After exposure, the cells were replenished with media containing the specific dose of each compound and incubated for 24 h. The nitrite production was measured in the supernatant of the cells using the Griess reagent system (Promega, USA) according to the manufacturer’s guidelines. The absorbance was measured at 540 nm wavelength using a spectrophotometer. The nitrite standard curve of nitrite concentration in µM was also produced using the 0.1 M nitrite standard provided with the kit. The concentration of nitrite generated in treated and control was calculated from the standard curve.

#### Determination of ROS generation and ROS scavenging activity

To measure the intracellular ROS, the fluorescent probe 2′,7′-dichlorodihydrofluorescein diacetate (H_2_DCFDA; Sigma-Aldrich, USA) was used. The BJ cells were seeded in a 96-well black fluorescence plate. After 24 h of incubation, the cells were washed gently with PBS and incubated overnight with the H_2_DCFDA probe (10 μM) in incomplete media. The next day after washing with PBS, cells were treated with compounds followed by UVA irradiation as before. After 24 h, a fluorescent spectrophotometer (Varioskan LUX, Thermo Fisher Scientific, USA) was used to measure the fluorescence of each well at an excitation and emission wavelength of 485 and 530 nm, respectively.

#### Detection of pro-inflammatory cytokines

To assess the presence of UVA-induced inflammation and inhibition of pro-inflammatory cytokines by the selected compounds, the presence of IL-1β and TNF-α in cell supernatant was determined by ELISA (Enzyme-Linked Immunosorbent Assay). The THP-1 cells were seeded at a seeding density of 2 × 10^5^ cells per well in a 24-well plate and incubated for 24 h. The following day RPMI medium containing 20 ng/mL of phorbol 12-myristate 13-acetate (PMA; Serva, Germany) was added to the THP-1 cells for 24 h, to allow differentiation of the cells into mature cytokine-producing macrophages. Afterward, the cells were treated with the EC_50_ concentration of the selected compounds and BP (reference compound) in PBS, followed by UVA irradiation. Next, the cells were replenished with RPMI medium-containing compounds and incubated further for 24 h. The supernatant was analyzed for the presence of cytokines using commercially available Human IL-1β and TNF-α ELISA kits (R&D Systems, Minneapolis, USA), according to the manufacturer's guidelines.

#### Assay for evaluation of DNA fragmentation

To determine the UVA-mediated fragmentation of DNA, agarose gel electrophoresis of the isolated DNA was performed. For this, the 85% confluent BJ cells were treated with compounds and were UVA-irradiated as mentioned previously. After exposure to UVA, the flask was replenished with fresh medium and incubated further for 5 h before isolation of genomic DNA. DNA was isolated using QIAGEN QIAamp^®^ DNA Mini and Blood Mini kit (Germany) according to the manufacturer’s guidelines. The isolated genomic DNA was analyzed on 1% agarose gel at 100 V for 120 min. The DNA bands in the gel were visualized under UV using the gel documentation system (ProteinSimple, FluorChem FC3, San Jose, CA, USA).

#### Immunofluorescence assay for detection of cyclobutane pyrimidine dimers (CPDs)

The formation of CPDs or thymine dimers by UVA irradiation in BJ dermal fibroblasts were detected by immunocytochemistry procedure, using an anti-thymine dimer antibody. For this, the cells were allowed to attach overnight to sterile coverslips. Next, the cells were treated with compounds and UVA and further incubated for 24 h. After incubation, cells were rinsed with 1X PBS and fixed with acetone (Sigma-Aldrich, USA) and methanol (Emplura, Sigma-Aldrich, USA) solution (1:1) for 20 min at room temperature (RT). Afterward, the acetone-methanol solution was aspirated and fixed cells were washed thrice with 1X PBS on an orbital shaker. Each wash was done for 3–5 min. For cell permeabilization, 0.1% Triton X-100 (Sigma-Aldrich, USA) in 1X PBS was added to each well for 10 min at RT followed by washing. For the retrieval of antigen within the chromatin, the cells were treated with a CPD antigen retrieval solution (70 mM NaOH dissolved in 70% ethanol in 1X PBS) for 30 min at RT. After washing, the blocking solution containing 2% BSA (Sigma-Aldrich, USA) and 0.1% Tween 20 (Sigma-Aldrich, USA) in 1X PBS was added and incubated at 37 °C for an hour in a pre-heated water bath. Next, 1:200 dilution of primary antibody (thymine dimer antibody; Novus Biologicals, CO, USA) was added to each well and left for overnight incubation at 4 °C. After washing, 1:200 dilution of fluorescent conjugated secondary antibody (Goat anti-Mouse IgG (H + L) Highly Cross-Adsorbed Secondary Antibody, Alexa fluor 568; Thermo Fisher Scientific, USA) prepared in blocking solution was added and incubated at 37 °C in a pre-heated water bath for 1 h. Again washing was done and 0.5 µg/mL DAPI (4′,6-diamidino-2-phenylindole; Thermo Fisher Scientific, USA) was added for 15 min at RT. The coverslips were mounted onto clean glass slides in presence of a mounting medium (Fluoromount-G; Thermo Fisher Scientific, USA) and allowed to dry before taking images. Images were taken using a fluorescence microscope (Ti-E, Nikon, Japan) with DS-Ri2 Nikon camera.

#### Statistical analysis

Statistical analysis of experimental data was performed by using the software IBM, Statistical Package for Social Sciences (SPSS, Chicago, USA), version 21. Both the IC_50_ and the EC_50_ values of each compound were calculated using Prism 6 (GraphPad Software Inc., California, USA) software using non-linear regression dose–response curve. The immunofluorescence images were analyzed using ImageJ software (National Institutes of Health, Bethesda, Maryland, USA). All experimental values were expressed as mean ± SEM (Standard Error of Mean). The statistical significance among the tested groups was evaluated by performing the one-way Analysis of Variance (ANOVA), followed by the Bonferroni post hoc test. A *p* value < 0.05 was considered statistically significant. In all graphs, ^###^*p* < 0.001 and ^##^*p* < 0.01 indicates a significant difference between the UVA group (UVA-irradiated cells) and the control group (untreated cells) while *p < 0.05, **p < 0.01, and ***p < 0.001 show a significant difference of the treatment groups (UVA + compound) upon comparison to the UVA-irradiated group (without compound).

## Results

### Cytotoxicity of compounds in a human dermal fibroblast cell line (BJ)

Cytotoxicity analysis was done to identify the compounds having an IC_50_ value higher than that of the reference. The reference compound (BP) showed an IC_50_ value of 53.4 ± 1.12 µM and compounds **DD-02** to **DD-04**, **DD-07**, **DD-09**, **DD-11**, **DD-14**, **DD-15**, and **DD-18** were found to have IC_50_ values greater than that of benzophenone (Table 1).

**Table 1:** In vitro cytotoxicity (IC_50_) of compounds (DD-01 to DD-19) and effective treatment dose (EC_50_) of selected compounds (having IC_50_ greater than that of the reference compound BP).

Compounds	Cytotoxic dose IC_50_ (µM ± SEM^b^)	Effective treatment dose EC_50_ (µM ± SEM^b^)
BP^a^	53.4 ± 1.12	3.692 ± 1.15
DD-01	46.95 ± 1.08	
DD-02	60.86 ± 1.21	5.541 ± 1.2
DD-03	60.39 ± 1.05	4.958 ± 1.2
DD-04	63.42 ± 1.04	4.876 ± 1.17
DD-05	48.34 ± 1.05	
DD-06	32.65 ± 1.14	
DD-07	75.35 ± 1.12	7.437 ± 1.2
DD-08	47.6 ± 1.09	
DD-09	81.45 ± 1.19	6.761 ± 1.2
DD-10	51.78 ± 1.05	
DD-11	62.99 ± 1.18	11.33 ± 1.06
DD-12	34.91 ± 1.12	
DD-13	48.03 ± 1.06	
DD-14	54.14 ± 1.04	4.497 ± 1.15
DD-15	81.98 ± 1.18	6.218 ± 1.19
DD-16	33.37 ± 1.12	
DD-17	43.89 ± 1.08	
DD-18	66.35 ± 1.08	21.54 ± 1.2
DD-19	28.59 ± 1.31	

^a^BP is the reference UV filter benzophenone. ^b^SEM is the standard error of the mean. All data were represented as mean ± standard error of the mean. Experiments were performed in triplicates (n = 3). For IC_50_ evaluation, the highest concentration was 100 µM and the IC_50_ concentration of each of the selected compounds was taken as the highest concentration for estimation of EC_50_. IC_50_ and EC_50_ values were calculated using Graph pad prism 6 software which was obtained using four concentrations of reference and test compounds.

### Selection of UVA dose

**Figure 2:**
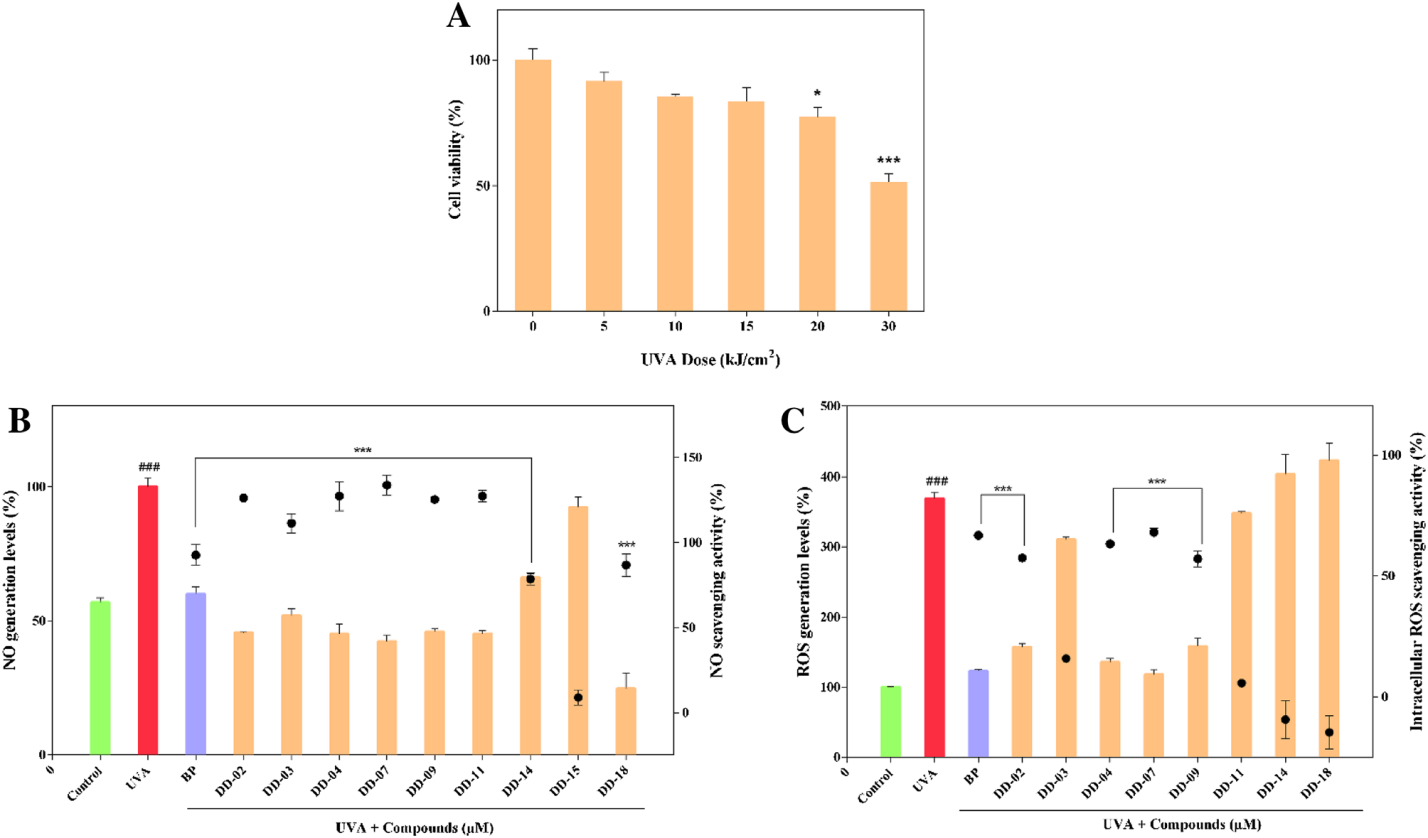
Effect of UVA irradiation on fibroblast cells. MTT cell viability assay was employed to assess the percentage of viable cells. Un-irradiated control (0 kJ/cm^2^) represent 100% cell viability (**A**). Detection of NO in UVA-irradiated fibroblast cells and effect of the EC_50_ concentration of compounds on the production of NO. The amount of NO (µM) was measured by Griess reagent assay following the manufacturer’s instructions. The bar graph represents the levels of NO generated (%) and the scatter plot shows the NO scavenging activity (%) of the compounds in presence of UVA (**B**). Detection of ROS in UVA exposed dermal fibroblast cells and the effect of an EC_50_ concentration of compounds on ROS generation and ROS scavenging activity. The production of ROS was measured by using H_2_DCFDA fluorescent probe. The bar graph showed the generation of ROS (%) and the scatter plot exhibited intracellular ROS scavenging activity (%) (**C**). All values are represented as mean ± S.E.M (n = 3).

MTT cell viability assay showed that UVA irradiation decreased the number of viable cells in a dose-dependent manner (Fig. 2A). The UVA dose of 20 kJ/cm^2^ (*p* < 0.05) and 30 kJ/cm^2^ (*p* < 0.001) significantly reduced the cell viability compared to control which was not exposed to UVA. At a UVA dose of 30 kJ/cm^2^, 51.4% cell viability was achieved and this dose was selected for further experiments.

### Effective treatment dose of compounds in presence of UVA irradiated BJ cells

The 9 compounds having an IC_50_ value greater than that of the BP (53.4 ± 1.12 µM) were selected for evaluation of EC_50_ treatment doses to be used in further assays. The EC_50_ value of BP was 3.692 ± 1.15 and the EC_50_ values of the selected compounds are mentioned in Table 1 along with their IC_50_ values.

### Effect of selected compounds on nitric oxide (NO) production in UVA exposed BJ cells

The EC_50_ concentration of BP and the selected compounds were tested for the reduction in NO. It was found that **BP, DD-02, DD-03, DD-04, DD-07, DD-09, DD-11, DD-14,** and **DD-18** significantly reduced the UVA-induced NO production (Fig. 2B). However, compound DD-15 did not show significant results and was not selected for subsequent experiments.

### Effect of selected compounds on intracellular ROS production in UVA irradiated BJ cells

The UVA-irradiated group showed a 369% increase in ROS levels compared to that of non-irradiated control cells. After UVA exposure, the intracellular ROS was significantly reduced in presence of **BP (reference compound), DD-02, DD-04, DD-07**, and **DD-09** (Fig. 2C). These compounds were selected for further assays.

### Effect of selected compounds on levels of pro-inflammatory cytokines in UVA irradiated THP-1 cells

**Figure 3:**
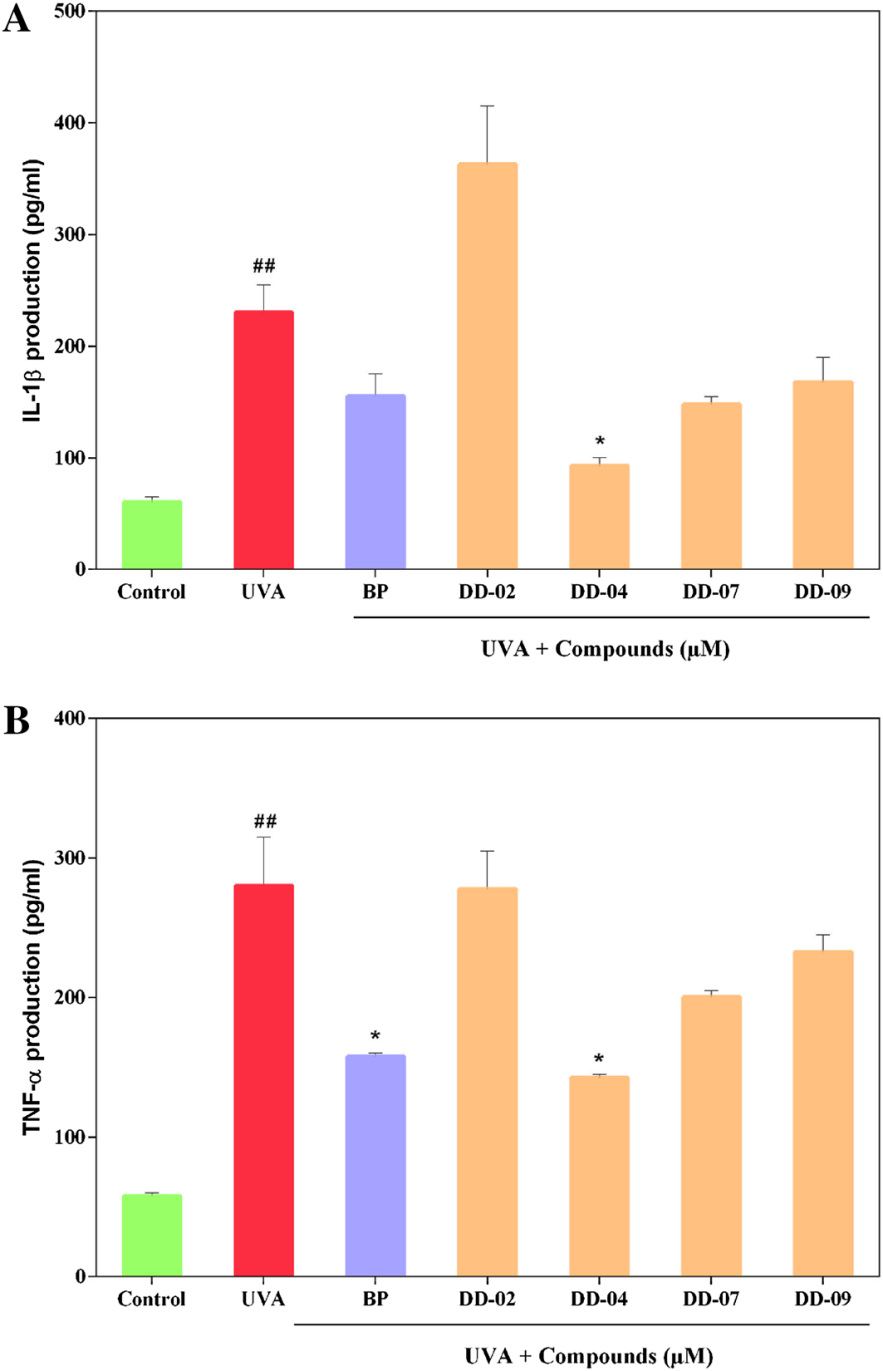
Detection of cytokine in UVA exposed THP-1 cells and their reduction in presence of compounds at EC_50_ concentration. The graph indicates the production of IL-1β in control and compound-treated groups (**A**). The bar graph showed the generation of TNF-α in control and compound treated groups (**B**). The production of pro-inflammatory cytokines was determined by using commercially available ELISA kits. The values are represented as mean ± S.E.M, where n = 2.

The production of IL-1β and TNF-α significantly increased in UVA-irradiated THP-1 cells compared to un-irradiated control cells. Production of both pro-inflammatory cytokines was significantly decreased in the presence of **DD-04** (Fig. 3).

### Treatment with DD-04 in presence of UVA shows normal morphology of BJ fibroblasts

**Figure 4:**
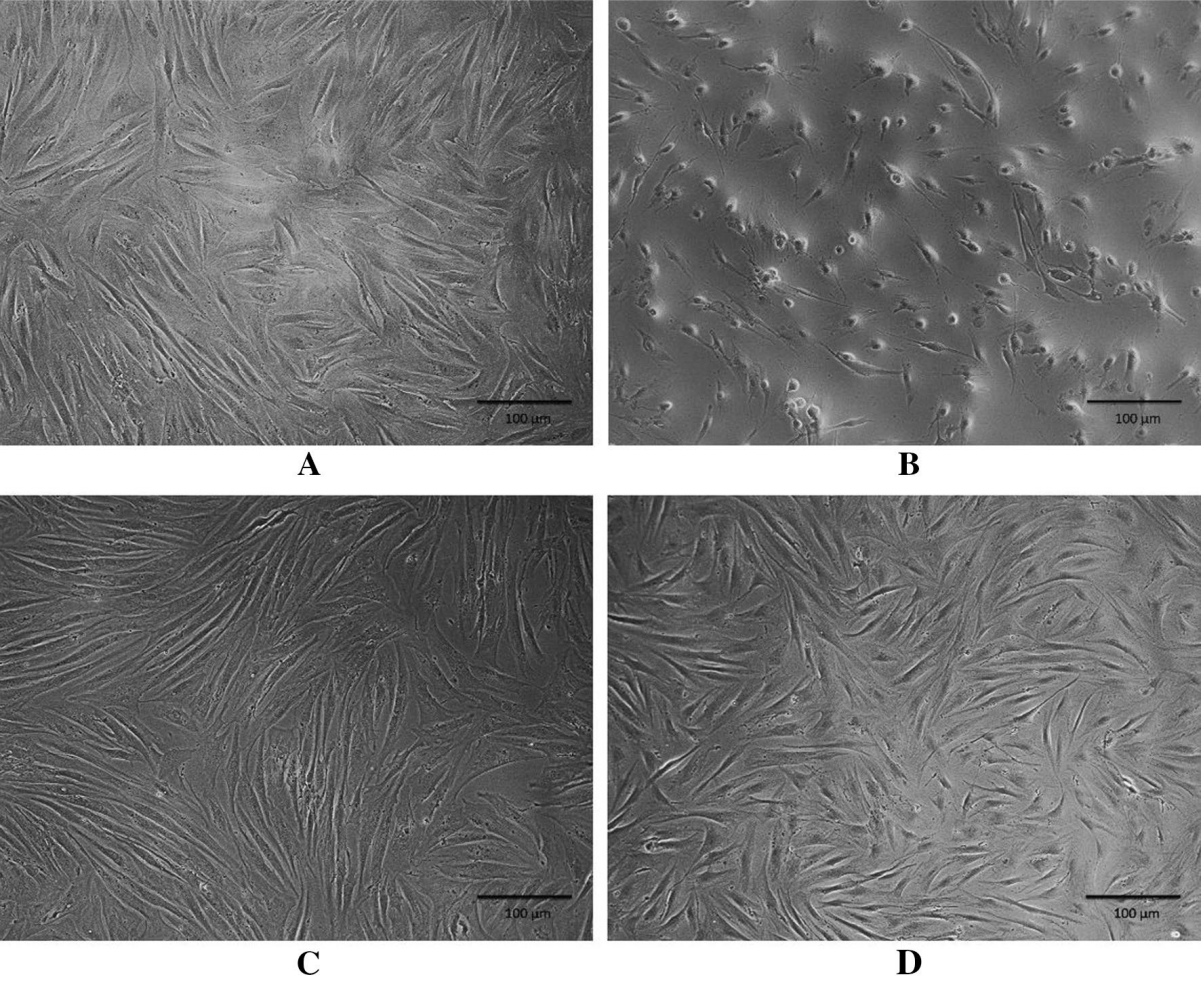
Morphological representation of human dermal fibroblasts (BJ cell line) using phase contrast microscopy. Morphology of normal fibroblast (control group) (**A**). Morphology of fibroblasts after UVA irradiation (**B**). Normal fibroblast morphology was observed after UVA irradiation in presence of the reference compound, BP (**C**). The morphology of **DD-04** treated fibroblasts after UVA irradiation showed similar morphology to the control and BP treated groups (**D**). Cells were observed at 20× objective. Unprocessed images of the cells are available in Fig. S1 in the supplementary file.

Different groups of BJ fibroblast cells (treated/non-treated) were observed for morphological changes (Fig. 4). The cells in UVA irradiated group appeared to have shrunken morphology (Fig. 4B) compared to the non-UVA irradiated control cells (Fig. 4A). The BP (Fig. 4C) and **DD-04** (Fig. 4D) treated group after UVA exposure showed normal fibroblast-like morphology. Hence, the compound **DD-04** showed promising protection from UVA-induced morphological changes in human dermal fibroblasts cells.

### DD-04 treatment in presence of UVA attenuated DNA fragmentation in BJ cells

**Figure 5:**
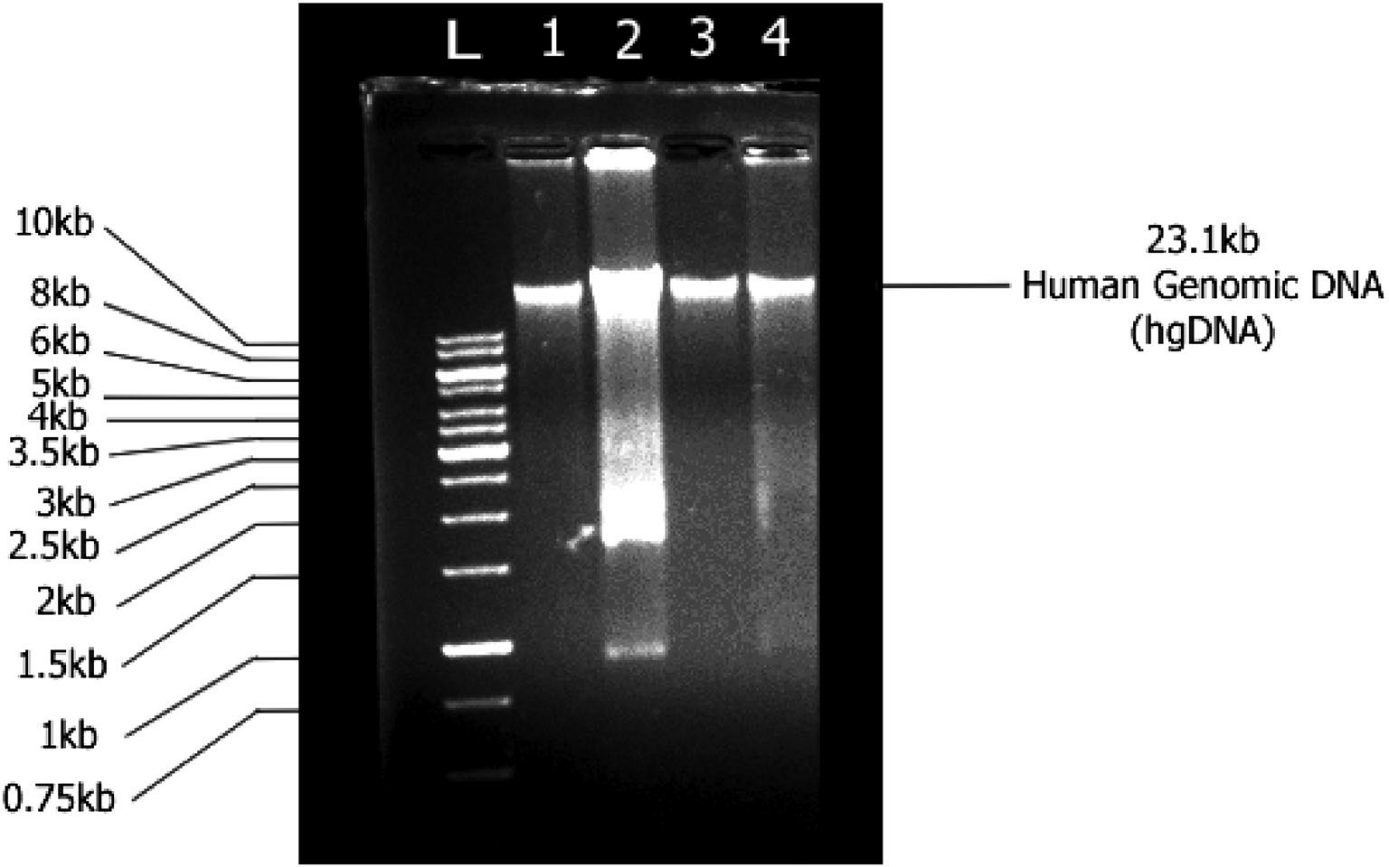
Effect of UVA on DNA fragmentation of BJ fibroblast cells. Lane 1, un-irradiated control cells (not treated with UVA and compounds); lane 2, UVA irradiated group; lane 3, UVA irradiated group in presence of BP; lane 4, UVA treated group in presence of **DD-04**. Lane L indicates a molecular DNA ladder (1 kb). DNA from the treated and the control cells were run on 1% agarose gel.

The DNA extracted from UVA-exposed cells showed a typical DNA fragmentation pattern (Fig. 5, lane 2). Whereas, DNA from un-irradiated control cells, which was run in lane 1, showed a single intact band indicating no damage to the DNA of cells. Similarly, cells treated with UVA in presence of the BP and **DD-04** showed a single intact band indicating their potential protective effect against UVA-induced DNA fragmentation (Fig. 5, lanes 3, 4, and 5).

### Detection of CPDs by immunocytochemistry

**Figure 6:**
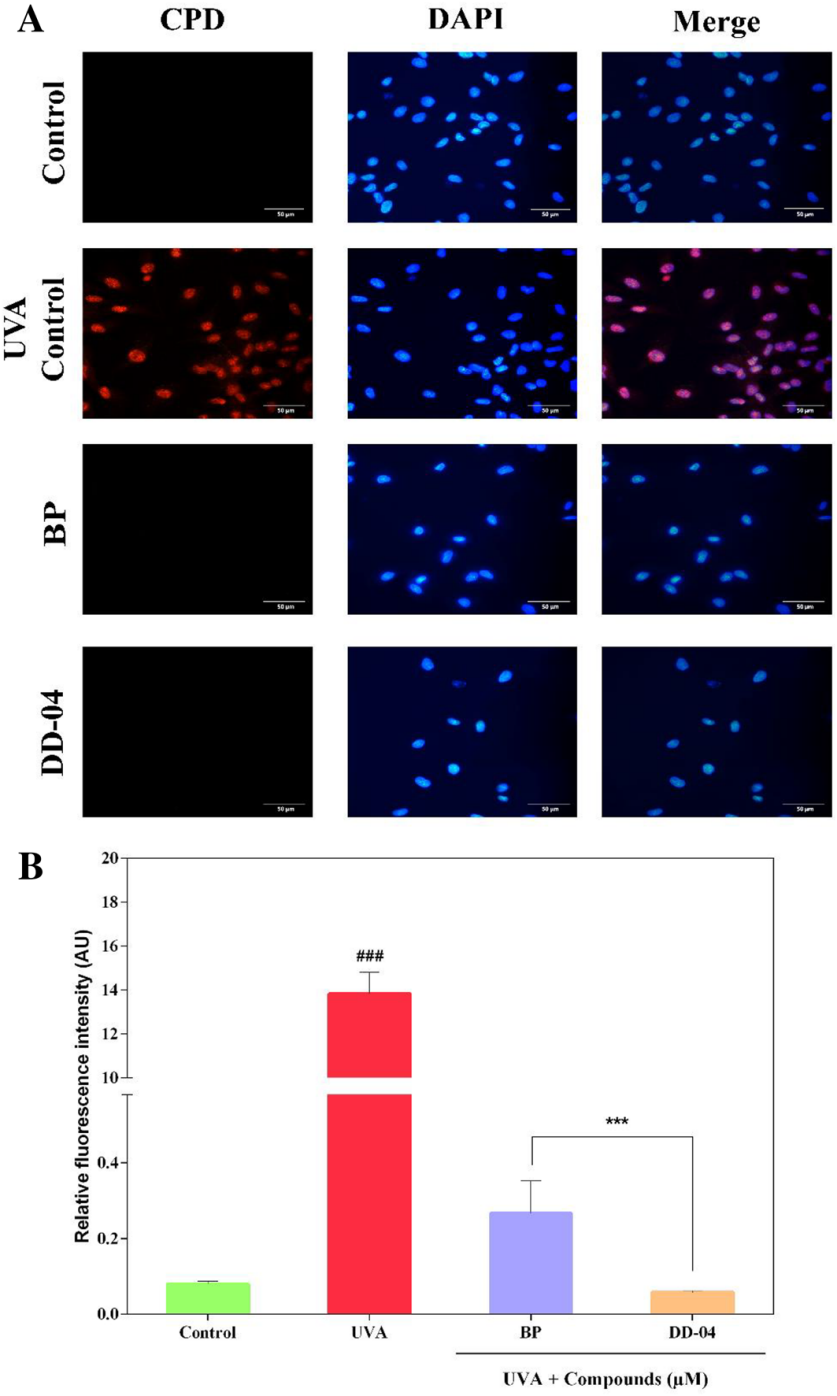
Immunocytochemical detection of CPD (thymine dimers) in UVA exposed fibroblast cells. The secondary antibody conjugated to Alexa fluor 568 was used for the detection of thymine dimers. Nuclei were counterstained with DAPI stain and cells were observed at 40 × objective. Thymine dimers were not detected in the control fibroblast cells without UVA exposure as seen in the immunofluorescence images, while the UVA control group showed a significant number of thymine dimers in the nucleus of the irradiated fibroblasts. The BP and **DD-04** group did not show any thymine dimers in the nucleus as well (**A**). The relative fluorescence intensity was calculated using ImageJ software, which revealed that both BP and **DD-04** treated groups showed a significant reduction in the thymine dimers compared to the UVA control group. The values are represented as mean ± S.E.M, where n = 3.

Immunocytochemical analysis was done to detect the presence of UVA-induced CPDs in exposed cells using antibodies specific to this type of DNA lesion. In the un-irradiated control group, the nuclei of cells remained unlabeled due to the absence of CPDs (Fig. 6A). Whereas in the UVA-irradiated group, a bright fluorescent signal was detected in the nuclei of the exposed cells. The compound treated group (BP and **DD-04**), after UVA irradiation, showed a significant reduction in fluorescent signal and the results were similar to that of un-irradiated control cells (Fig. 6B).

## Discussion

UVA is now a known carcinogen but it was not given clinical importance until the first UVA filter was introduced in 1980. Later in 1992, the star rating system was developed to ensure sunscreen rating for UVA protection similar to the Sun Protection Factor (SPF), which is a UVB protection rating system for marketed sunscreens^35^. Currently, as of February 2019, FDA has approved only 2 UV filters (zinc oxide and titanium dioxide) in Category I of GRASE (Generally Recognized As Safe and Effective) that can be used in sunscreens. The FDA also enlists 12 marketed UV filters in GRASE Category III which require further toxicological studies. Out of these, benzophenone, avobenzone, oxybenzone, sulisobenzone, octisalate, octinoxate, octocrylene, cinoxate, and homosalate are the famous UVA filters^36^. Thus, considering the current escalation in skin cancer cases, there is a greater need to develop newer UVA filters with effective photoprotection and a safer toxicological profile^37^.

The current study was proposed to examine the in vitro photoprotection and cytotoxicity of the lipophilic chain-linked thiourea derivatives. Substantial literature supports the fact that thiourea is a potent antioxidant and has a high degree of free radical scavenging potential^38^. This antioxidant capacity of thiourea is directly linked to its anti-inflammatory and anti-cancer properties, as depicted in various studies^33,39,40^. Research has also shown that thiourea derivatives prevented the formation of melanoma skin cancer (anti-melanoma activity)^32,41^. Another study revealed that thiourea derivatives are also antimicrobial (antibacterial and antifungal) in nature^31^. Upregulated levels of MMPs are found in UVA-exposed skin and are linked to the degradation of ECM^13^. A study showed that thiourea analogue inhibited the production of MMP enzymes via a decrease in the production of TNF-α^33^. All these properties of thiourea derivatives make it a perfect candidate compound to study for its photoprotective potential against UVA radiation exposure, which to our knowledge has not yet been studied.

Apart from the thiourea moiety, the lipophilic chain in the structure of compounds also serves an important purpose. Since UVA is highly penetrable and can easily reach the dermis region of the skin, the thiourea moiety was attached to a lipophilic chain to ensure that the compounds reach the dermis as well^42^. The stratum corneum is the top layer of the skin which provides a barrier to hydrophilic molecules. Moderately lipophilic molecules penetrate this layer via the transcellular route. Any compound or drug that is highly lipophilic or hydrophilic is poorly absorbed through the skin^43^. Thus, moderation in the hydrophilic-lipophilic balance (HLB) is necessary for designing a good chemical UV filter in any formulation^44^. The compounds under study have a lipophilic chain at one end and a hydrophilic structure at the other end, making them a good HLB molecule to study for UV protection.

The first and foremost step in the development of a new UV filter is to determine its toxic effect on both in vitro and in vivo model systems before moving toward the human trial^45^. Measuring the cytotoxic effect of compounds by using MTT dye is considered a gold standard^46^. The normal human fibroblasts cell line in our study was treated with various concentrations of the compounds and cell viability was assessed. The compounds, in comparison to the reference compound, that did not show any significant cytotoxic effect were selected. Furthermore, the concentration of selected compounds that protected the cells from UVA-induced cell death was determined as well. Thus, the concentration of compounds which was non-cytotoxic in presence of UVA was used in the proceeding experiments.

UVA is regarded as the major extrinsic factor leading to the production of nitric oxide by dermal fibroblasts and other resident dermal cells^16^. Highly toxic free radical derivatives of NO like peroxynitrite acts as potent nitrative agents that damage the DNA bases, in which nitration of guanine to 8-nitroguanine is most prominent^47,48^. The 8-nitroguanine is highly promutagenic as it undergoes rapid depurination to form abasic sites. These abasic sites lead to loss of genomic integrity by accumulating mutations within the DNA^49^. NO is also involved in the inhibition of various DNA repair enzymes like 8-oxoguanine glycosylase (OGG1), which readily removes DNA lesions that contribute to carcinogenesis^49–51^. Nitrous anhydride (N_2_O_3_), a derivative of NO is also primarily responsible for the formation of DNA strand breaks^47^. UVA-induced overproduction of NO also interferes with the excision and ligation steps of NER of CPDs^52,53^. Considering the important role of NO in UV-induced pathology, the present study investigated the role of our test compounds in reducing UVA-induced NO. NO production was higher in UVA-exposed cells and was lowered by the addition of compounds. The lower level of NO production may depict inhibition of the iNOS enzyme at the molecular level by the compounds.

The compounds showing promising inhibition of NO were tested for their ability to reduce oxidative damage by ROS. As mentioned before, ROS generation is the hallmark of UVA-induced damage to cells. UVA photons cause photosensitization of chromophores which is the root cause of ROS generation^54^. UVA from either artificial or natural sources is linked to the formation of melanomagenesis^55^. Through various studies, it is evident that the accumulation of cellular and genomic oxidative damage via UVA-induced sensitization of melanin is involved in MM formation^22,56^. Moreover, ROS generated by UVA forms various types of DNA lesions, including the 8-oxo-7,8-dihydroguanine (8-oxoG), genomic strand breaks, formation of abasic sites, and DNA–protein crosslinks^57,58^. UVA-induced ROS also causes oxidation of DNA repair enzymes rendering them unable to repair DNA damages^59^. Thus, suppression and elimination of UVA-induced ROS are of key importance when designing a new UVA filter. In the current study, ROS generation and ROS scavenging activities were evaluated. The tested compounds that showed promising inhibition of ROS in presence of UVA were selected for evaluation of pro-inflammatory cytokines.

Acute exposure to solar UV radiation causes dermal inflammation, in which neutrophils and macrophages play an important role along with keratinocytes and fibroblasts. IL-1 β and TNF-α are the primary pro-inflammatory cytokines known to commence the inflammatory response and also induce the production of other inflammatory cytokines^60^. Neutrophils are the first immune cells to infiltrate the epidermis and dermis region of skin after UV exposure. Their function is to clear the apoptotic cells and cells with oxidized surface lipids^61^. Next, macrophages produce various ECM degrading proteolytic enzymes like MMPs, elastase, collagenase, etc. at the inflamed site^62^. Repeated exposure to UV radiation causes frequent macrophage infiltration which destroys the integrity of ECM and hampers the ECM repair function of dermal fibroblasts as well. Macrophages also produce large quantities of ROS after UV exposure^63^. NF-κB transcription factor plays a central role in the induction of various inflammatory diseases and cancer. NF-κB can be activated by ROS generated by UV irradiation of the dermal region. This activation of NF-κB triggers the upregulation of pro-inflammatory cytokine expression in immune cells including monocytes and macrophages^15^. IL-1 β and TNF-α are the key cytokines produced by monocytes and activated macrophages after UV irradiation^64^. NF-κB also induces the production of proteolytic enzymes (like MMP) and NO via activation of the iNOS enzyme^65^. To identify the anti-inflammatory effect of the selected compounds, we studied the UVA-induced production of pro-inflammatory cytokines (IL-1β and TNF-α) in the macrophage model cell line, THP-1. We observed that the production of both pro-inflammatory cytokines increased significantly upon UVA exposure and decreases significantly upon treatment with the compound **DD-04**. These results (along with the aforementioned results) indicate that **DD-04** has promising antioxidant and anti-inflammatory activity. It can also be suggested that **DD-04** suppressed the activation of NF-κB, which in turn downregulated the expression of iNOS, IL-1β, and TNF-α.

**Figure 7:**
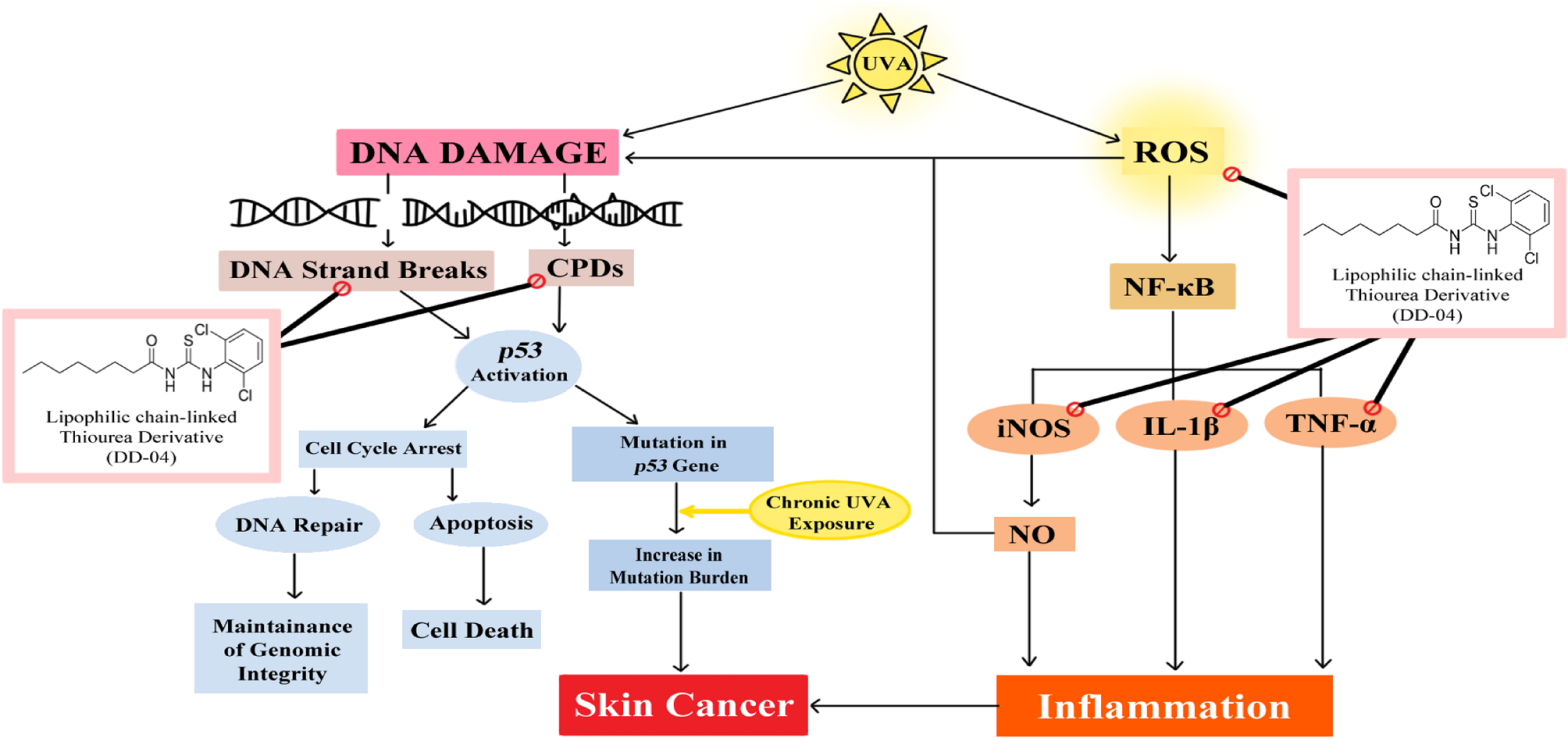
Proposed mechanism for attenuation of UVA-induced photocarcinogenesis and inflammation by lipophilic chain-linked thiourea derivative (**DD-04**).

Since **DD-04** showed good activity against ROS and inflammation, it was further tested for its protective potential against the formation of UVA-induced CPDs (thymine dimers) and DNA strand breaks. CPDs are the most common and highly mutagenic bulky modification that occurs within the DNA upon UV exposure. Although the absorption of UVA wavelength is 4.2-fold less than that of UVB, it is still responsible for the generation of CPDs. This is mainly due to the 20-fold higher incidence of UVA compared to UVB on earth^1^. Studies have also shown that UVA radiation-induced CPDs are more mutagenic than CPDs formed by UVB radiation. This is due to less effective activation of anti-mutagenic DNA damage responses which subsequently results in replication of DNA with damages^66^. Moreover, it is also worth noting that UVA radiation being highly penetrable can mutate the stem cell pool of the dermal region^67^. UVA is also responsible for the formation of dark CPDs which are formed through chemiexcitation via ROS and RNS. These CPDs are formed long after exposure to UV radiation^68^. Another type of damage induced by UVA is the DNA strand breaks which are formed via intermediates of ROS generated by UV exposure. According to a study, the use of an antioxidant (naringin) prevented the formation of strand breaks after UVA exposure in vitro^28^. In our study, we examined the preventive role of **DD-04** against UVA-induced DNA strand breaks by agarose gel electrophoresis. The typical DNA smearing was observed in UVA irradiated group while UVA irradiation in presence of **DD-04** revealed a single band, which indicates that no fragmentation or strand break has occurred. Finally, anti-thymine dimer antibody was used to detect the formation of CPDs by immunocytochemistry. The results showed absence of thymine dimers in **DD-04** treated group compared to the UVA exposed group. Overall, our results indicate that the mechanism by which lipophilic chain-linked thiourea derivative (**DD-04**) inhibits UVA-induced inflammation and photocarcinogensis may be linked to its antioxidant property which inhibited ROS, NO, and formation of CPDs and strand breaks within DNA (Fig. 7).

## Conclusion

In conclusion, **DD-04** showed good ROS and RNS scavenging potential which correlates to its protective potential against UVA-induced cellular damages, including inflammation, formation of CPDs, and strand breaks in DNA. Therefore, lipophilic chain-linked thiourea derivative (**DD-04**) can be an excellent UVA filter to prevent UVA-related skin problems including prevention of skin malignancies and photoaging. However, it can be further corroborated by conducting in vivo studies as well.

## Supplementary Information

Supplementary Figure S1.

## Data Availability

All data generated or analyzed during this study are included in this published article and its supplementary information files.
