# Supplementary material for: Probing photoprotection properties of lipophilic chain conjugated thiourea-aryl group molecules to attenuate ultraviolet-A induced cellular and DNA damages
Source: Sci Rep. 2022 Dec 3;12:20907. doi: 10.1038/s41598-022-25515-5 (PMC9719470; doi:10.1038/s41598-022-25515-5)
Supplement: Supplementary file 1 — Supplementary Figure S1. [file 41598_2022_25515_MOESM1_ESM.pdf]

Supplementary information file

Probing Photoprotection Properties of Lipophilic Chain Conjugated Thiourea-Aryl Group Molecules to Attenuate Ultraviolet-A Induced Cellular and DNA Damages

Sobia Rana[a*], Noor Fatima[a], Sana Yaqoob[b], Abdul Hameed[b,c*], Munazza Raza Mirza[a], Almas Jabeen[a], Jamshed Iqbal[d]

[a]*Molecular Biology and Human Genetics Laboratory, Dr. Panjwani Center for Molecular Medicine and Drug Research (PCMD), International Center for Chemical and Biological Sciences (ICCBS), University of Karachi, Karachi-75270, Pakistan*

[b]*H. E. J. Research Institute of Chemistry, International Center for Chemical and Biological Sciences, University of Karachi, Karachi, Pakistan*

[c]*Department of Chemistry, University of Sahiwal, Sahiwal, Pakistan*

[d]*Center for Advanced Drug Research, COMSATS University Islamabad, Abbottabad Campus, Abbottabad 22060, Pakistan*

*Dr. Sobia Rana (Corresponding Author)

Molecular Biology and Human Genetics Laboratory, Dr. Panjwani Center for Molecular Medicine and Drug Research (PCMD), International Center for Chemical and Biological Sciences (ICCBS), University of Karachi, Karachi-75270, Pakistan

https://orcid.org/0000-0003-2540-5170

molecularbiologist1@gmail.com;sobia.rana@iccs.edu

+ 92 21 99261683

*Abdul Hameed (Corresponding Author)

H. E. J. Research Institute of Chemistry, International Center for Chemical and Biological Sciences, University of Karachi, Karachi, Pakistan.

Department of Chemistry, University of Sahiwal, Sahiwal, Pakistan

Abdul_hameed8@hotmail.com

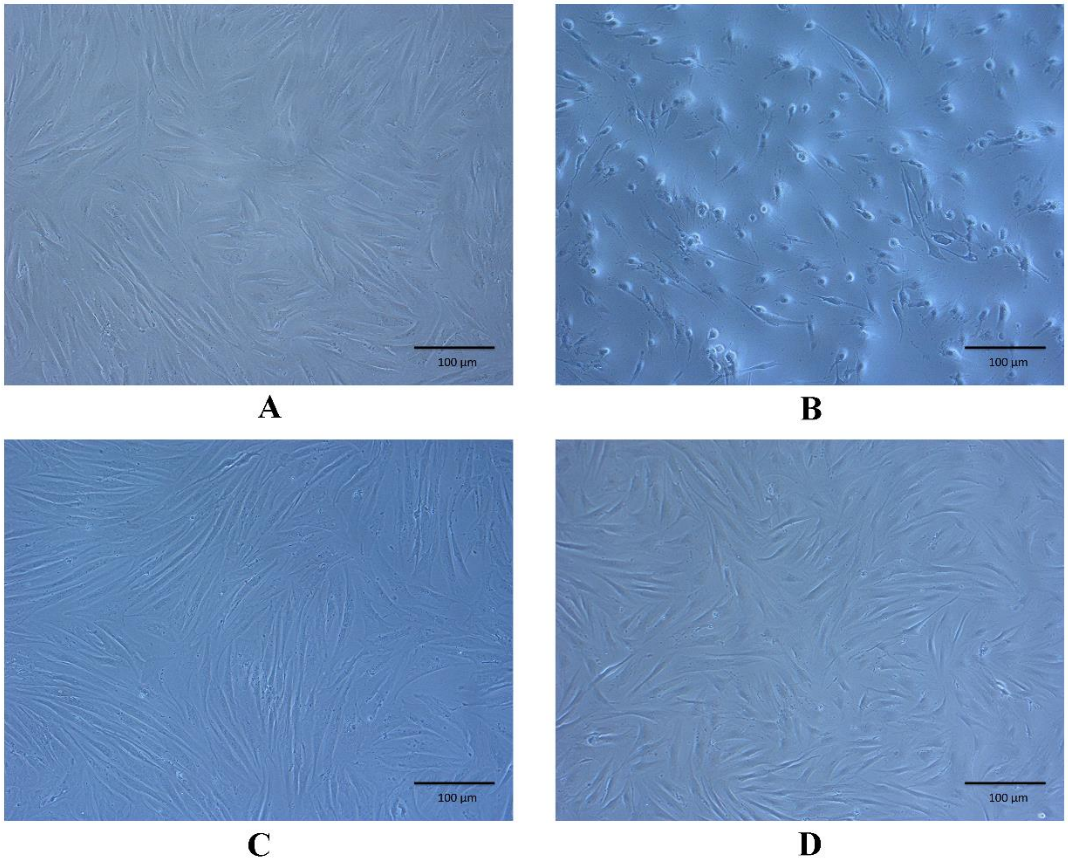

Figure S1 Morphological representation of human dermal fibroblasts (BJ cell line) using phase contrast microscopy.

Morphology of normal fibroblast (control group) (A). Morphology of fibroblasts after UVA irradiation (B). Normal fibroblast morphology was observed after UVA irradiation in presence of the reference compound, BP (C). The morphology of DD-04 treated fibroblast after UVA irradiation showed similar morphology to the control and BP treated group (D). Cells were observed at 20X objective.
